# *PCSK9* variation and association with blood pressure in African Americans: preliminary findings from the HyperGEN and REGARDS studies

**DOI:** 10.3389/fgene.2015.00136

**Published:** 2015-04-08

**Authors:** Ngan T. Tran, Stella Aslibekyan, Hemant K. Tiwari, Degui Zhi, Yun Ju Sung, Steven C. Hunt, DC Rao, Ulrich Broeckel, Suzanne E. Judd, Paul Muntner, Shia T. Kent, Donna K. Arnett, Marguerite R. Irvin

**Affiliations:** ^1^Department of Epidemiology, University of Alabama at BirminghamBirmingham, AL, USA; ^2^Department of Biostatistics, University of Alabama at BirminghamBirmingham, AL, USA; ^3^Department of Biostatistics, Washington University in St. LouisSt. Louis, MO, USA; ^4^Department of Internal Medicine, University of UtahSalt Lake City, UT, USA; ^5^Department of Medicine, Human and Molecular Genetics Center, Medical College of WisconsinMilwaukee, WI, USA

**Keywords:** *PCSK9*, blood pressure, hypertension, dyslipidemia, low-density lipoprotein cholesterol

## Abstract

Proprotein convertase subtilisin/kexin type 9 (encoded by *PCSK9*) plays a well-known role in the regulation of low-density lipoprotein (LDL) receptors, and an inhibitor of this enzyme is a promising new therapeutic for hyperlipidemia. Recently, animal and human studies also implicate *PCSK9* genetic variation in the regulation of blood pressure. The goal of this study was to examine if common and rare polymorphisms in *PCSK9* are associated with blood pressure in an African-American population at high risk for cardiovascular disease. Using genomic data assayed on the Affymetrix 6.0 array (*n* = 1199) and the Illumina HumanExome Beadchip (*n* = 1966) from the Hypertension Genetic Epidemiology Network (HyperGEN), we tested the association of *PCSK9* polymorphisms with blood pressure. We used linear mixed models and the sequence kernel association test (SKAT) to assess the association of 31 common and 19 rare variants with blood pressure. The models were adjusted for age, sex, center, smoking status, principal components for ancestry and diabetes as fixed effects and family as a random effect. The results showed a marginally significant effect of two genome-wide association study (GWAS) single-nucleotide polymorphisms (SNPs) (rs12048828: β = 1.8, *P* = 0.05 and rs9730100: β = 1.0, *P* = 0.05) with diastolic blood pressure (DBP); however these results were not significant after correction for multiple testing. Rare variants were cumulatively associated with DBP (*P* = 0.04), an effect that was strengthened by restriction to non-synonymous or stop-gain SNPs (*P* = 0.02). While gene-based results for DBP did not replicate (*P* = 0.36), we found an association with SBP (*P* = 0.04) in the Reasons for Geographic And Racial Differences in Stroke study (REGARDS). The findings here suggest rare variants in *PCSK9* may influence blood pressure among African Americans, laying the ground work for further validation studies.

## Introduction

Hypertension is currently a major health problem in African Americans (Kramer et al., 2004). Data from the National Health and Nutrition Examination Survey (NHANES) in 2008 showed that age-adjusted prevalence of hypertension was 40% in African Americans vs. 30% in Caucasians (Egan et al., 2010). Overall, African Americans are more prone to hypertension-related morbidity and mortality than Caucasians (Lackland, 2014). For instance stroke risk is twice as high and risk for end-stage renal disease is 4–5 times higher among African Americans (Go et al., 2014). The reason for this disparity is not completely understood, although differences in genetic background are hypothesized to play a role (Allison et al., 1994; Kato, 2012). Because dyslipidemia is linked to hypertension, genetic variants associated with lipid levels represent promising candidates for novel hypertension loci (Brown et al., 2000; Sesso et al., 2005; Halperin et al., 2006; Nguyen et al., 2008; Maharjan et al., 2012; Yin et al., 2012).

Proprotein convertase subtilisin/kexin type 9 (encoded by the *PCSK9* gene) contributes to low-density lipoprotein (LDL) cholesterol levels by reducing cell surface expression of LDL receptors. Mutations in *PCSK9* can cause hypercholesterolemia (gain of function) and hypocholesterolemia (loss of function), which has made it an attractive therapeutic target for the treatment of dyslipidemia (Horton et al., 2007; Abifadel et al., 2009). Recently, research in an animal model suggested a role for *PCSK9* in the regulation of blood pressure (Sharotri et al., 2012), showing that the *PCSK9* gene product decreases trafficking of the epithelial sodium channel (ENaC) protein to the cell surface in *Xenopus* oocytes by enhancing proteasomal degradation. ENaC forms a pathway for sodium to enter epithelial cells at the apical membrane, thus regulating sodium absorption in the kidney and contributing to blood pressure regulation. Therefore, *PCSK9* activity could affect blood pressure by modulating sodium absorption. Because most previous studies of *PCSK9* have focused on cholesterol metabolism, the role of *PCSK9* in blood pressure is a novel area of investigation. To address this gap, we examined the association of common and rare variants in *PCSK9* and blood pressure in a population of African Americans at high risk for cardiovascular disease.

## Methods

### Study population

We used clinical and genetic data collected from African Americans participating in the Hypertension Genetic Epidemiology Network (HyperGEN) study. HyperGEN was designed to study genes promoting hypertension as part of the Family Blood Pressure Program funded by the National Heart, Lung and Blood Institute. African-American participants were recruited in Forsyth County, NC and Birmingham, AL from siblings who were diagnosed with hypertension before age 60 years and had a least one additional hypertensive sibling who agreed to participate. In the second phase of the study, the offspring of the original sib-pairs were recruited. The HyperGEN study was approved by Institutional Review Boards of the collaborating institutions. All participants gave written informed consent.

Participants with type 1 diabetes or advanced renal disease (defined as serum creatinine level > 2 mg/dL) were excluded from the parent HyperGEN study since these two conditions can cause secondary hypertension and the focus of HyperGEN was to identify novel essential hypertension loci. Patients taking lipid-lowering agents or missing lipid-lowering treatment data were excluded from the current analysis (*n* = 59 from the common variant analysis and 145 in the rare variant analysis).

### Clinical variables

Six consecutive BP measurements were made during the study visit, three on the left and three on the right arm. The participants rested for 5 min before measurement and for 30 s between measurements (Knox et al., 2010). An average sitting systolic blood pressure (SBP) and diastolic blood pressure (DBP) was calculated based on the second and third measurements in the second arm. Blood pressure measurements were obtained using automated Dinamap devices (model 1846 SX/P, Critikon, Tampa, FL) to promote comparable measurements among HyperGEN field centers. Hypertension was defined as average SBP ≥ 140 mm Hg or average DBP ≥ 90 mm Hg, or using at least one class of antihypertensive medication. Participants were categorized as treated if they were taking at least one antihypertensive medication according to the Seventh Report of the Joint National Committee on Prevention, Detection, Evaluation, and Treatment of High Blood Pressure (JNC 7) classifications (Chobanian et al., 2003). Participants were categorized as having diabetes if they were on anti-diabetic medications or insulin treatment, or had fasting glucose ≥126 mg/dl.

More details regarding study design and methodology of the HyperGEN study are available in previous publications (Williams et al., 2000).

## Genotyping

### Common variants

DNA extraction and purification from stored blood were performed using commercial Puregene reagents (Gentra System, Inc., Minneapolis, MN) as previously described (Williams et al., 2000). Genome-wide association study (GWAS) genotyping was performed using the Affymetrix Genome-Wide Human SNP 6.0 Array following the Affymetrix defined protocol (Arnett et al., 2011). Using a subset of 1258 subjects, approximately 3.01 million HapMap SNPs were imputed using MACH v. 1.0 with Human Genome Build 36 as the reference. Overlapping SNP genotypes were 99.5% concordant in the imputed dataset as compared to the genotype data set. SNPs which were not in HapMap (*n* = 94.337), monomorphic (*n* = 14.363), *R*^2^ < 0.3, minor allele frequency (MAF) < 1%, or Hardy-Weinberg equilibrium (HWE) *P* ≤ 10^−6^ (*n* = 150.096) were removed from the imputed dataset. The overall Mendelian error rate was 0.045%. A total of 50 common variants in *PCSK9* were obtained from the imputed GWAS data in 1199 samples. Using Haploview (Barrett et al., 2005) with *r*^2^ threshold at 0.8 to examine linkage disequilibrium between the 50 common SNPs (Supplementary Material), we chose 31 independent tag SNPs in *PCSK9* for our analysis. We additionally considered the associations of 3 SNPs located near *PCSK9* that were reported in prior GWAS of lipid traits [rs11206510 (Schunkert et al., 2011), rs2479409 (Willer et al., 2013), and rs2495478 (Turnbull et al., 2012)] with both SBP and DBP.

### Rare variants

Study samples were processed on the HumanExome BeadChip v1.0 (Illumina, Inc., San Diego, CA) using manufacturer protocols for a total of 2111 samples. The total number of SNPs assayed was 242,901. Monomorphic SNPs (*n* = 105.723), SNPs with missing rate >5% (*n* = 36), and SNPs with Hardy-Weinberg equilibrium *P* < 10^−6^ (*n* = 175) were removed. The Mendelian error rate was 0.002%. A total of 19 rare variants (MAF < 0.01) in *PCSK9* were included in this analysis from 1966 samples. There were 1131 samples that had both common and rare variant data available for *PCSK9* from GWAS and exome chip assays, respectively.

### Statistical methods

To reduce bias and improve statistical power, the effect of antihypertensive treatment was controlled by adding 15 mm Hg to observed SBP and 10 mm Hg to DBP for those who reported taking medications (Tobin et al., 2005). To control for population substructure 10 principal components were generated in Eigenstrat (Price et al., 2006).

For common *PCSK9* variants, we used linear mixed models adjusted for age, sex, center, smoking status, principal components, diabetes (fixed effects), and family (random effect) to evaluate main genetic effects. For rare variants, we used the sequence kernel association test (SKAT) (Wu et al., 2011) adjusting for the same covariates. We also examined the joint effect of common and rare variants in PCSK9 with blood pressure among the subset with both GWAS and exome chip data (*n* = 1131) using SKAT. Common variants that achieved nominal statistical significance (*P* < 0.05) in the mixed models were selected to test the joint effect.

A *P*-value of 0.05 was considered as suggestive evidence for an association. A Bonferroni correction was used to correct for multiple tests of common variants, where the type 1 error rate was equal to 0.05/31 = 0.0016. R version 3.0.2 was used for SKAT and SAS version 9.3 (SAS Institute Inc., Cary, NC, USA) was used for all other analyses.

### Replication

Results from HyperGEN reaching at least marginal significance (*P* ≤ 0.05) were tested in The REasons for Geographic And Racial Differences in Stroke (REGARDS) Study. REGARDS is a national, population-based, longitudinal study of incident stroke and associated risk factors among 30,239 African-American and European-American adults aged ≥ 45 years. Participants were randomly sampled and contacted by mail, then telephone, and enrolled between 2003 and 2007. A total of 7700 randomly selected African Americans were recently genotyped with the same Illumina exome chip as HyperGEN. Similar QC as in HyperGEN was carried out (Howard et al., 2005). There was a total of 1502 participants with GWAS data and 4960 participants with exome chip data after we excluded those with missing information on blood pressure or antihypertensive treatment and those on lipid-lowering drugs. We used similar statistical methodology and adjustment for covariates including ancestry to examine the association of selected variants with blood pressure in REGARDS. The REGARDS study was approved by Institutional Review Boards of the collaborating institutions and all participants gave written informed consent.

## Results

Table 1 shows the baseline characteristics of HyperGEN participants with *PCSK9* variant data available from GWAS, the exome chip, and both assays. The majority of participants were middle-aged and female, as well as obese (BMI ≥ 30 kg/m^2^) and hypertensive. Diabetes prevalence did not exceed 17% in any subset. The REGARDS population was also mostly female (64%), but was, on average, older (mean age 63.9 ± 9) with a higher prevalence of diabetes (24.5%).

**Table 1:** **Baseline characteristics for African-American participants with exome chip data, GWAS data, and both data sets**.

	**Exome chip (*N* = 1966)**		**GWAS (*N* = 1199)**		**Both (*N* = 1131)**	
	***N***	**%**	***N***	**%**	***N***	**%**
**AGE, YEARS**						
<30	226	11.4	178	14.9	161	14.2
30–60	1431	72.2	849	70.8	802	70.9
60+	326	16.4	172	14.3	168	14.9
**SEX**						
Male	729	36.8	393	32.8	368	32.5
Female	1254	63.2	806	67.2	763	67.5
**CENTER**						
Forsyth County, NC	495	24.9	247	20.6	223	19.7
Birmingham, AL	1488	75.1	952	79.4	908	80.3
**SMOKING**						
Never	979	50.2	618	52.0	570	50.8
Ever	971	49.8	571	48.0	552	49.2
**CURRENT SMOKING**						
No	1392	71.4	862	72.5	808	72.0
Yes	558	28.6	327	27.5	314	28.0
**DBP, mm Hg**						
<90	1791	90.3	1086	90.6	1022	90.4
≥90	192	9.7	113	9.4	109	9.6
**SBP, mm Hg**						
<140	1434	72.3	863	72.0	815	72.1
≥140	549	27.7	336	28.0	316	27.9
**HYPERTENSION**						
No	822	41.4	470	39.2	433	38.3
Yes	1161	58.6	729	60.8	698	61.7
**DIABETES**						
No	1648	83.1	1005	83.8	945	83.6
Yes	335	16.9	194	16.2	186	16.4
**BMI, kg.m^2^**						
<25	124	6.3	98	8.2	90	8.0
25–29.9	102	5.1	80	6.7	71	6.3
30+	1757	88.6	1021	85.1	970	85.7

*BMI, body mass index; DBP, diastolic blood pressure; GWAS, Genome-wide association study; SBP, systolic blood pressure*.

All SNPs were in HWE except for rs17405810 (HWE *P* = 0.04). Table 2 shows the association of the 31 common SNPs with blood pressure. The SNPs rs12048828 and rs9730100 were both marginally associated with DBP (both *P* = 0.05). There were no significant associations observed with SBP. The two GWAS SNPs did not show significant association with DBP (*P* = 0.44 for rs4927219 and 0.89 for rs12048828) or SBP (*P* = 0.19 and 0.42, respectively) in the REGARDS population (rs4927219 was used as a proxy for rs9730100) (Ward and Kellis, 2012). Our look-up of 3 known lipid loci near *PCSK9* from prior GWAS showed no significant association with DBP or SBP [rs11206510 (*P* = 0.3 for DBP and 0.8 for SBP), rs2479409 (*P* = 0.1 for DBP and 0.4 for SBP), and rs2495478 (*P* = 0.7 for DBP and 0.6 for SBP)]. Table 3 annotates 19 rare variants in *PCSK9* (MAF ≤ 0.01) included in this study. Overall, we observed a higher median SBP and DBP for carriers of non-synonymous SNPs (Table 3) compared to non-carriers (median SBP and DBP for non-carriers is 127 mm Hg and 73 mm Hg, respectively). Association analysis demonstrated a significant cumulative effect of rare variants with DBP (*P* = 0.04) but not with SBP (*P* = 0.14) in HyperGEN. We separately tested the association of 16 non-synonymous or stop-gain SNPs and 3 synonymous SNPs with DBP where there was a significant cumulative effect of non-synonymous or stop-gain SNPs (*P* = 0.02) but not synonymous SNPs (*P* = 0.73). The joint effect of rs12048828, rs9730100, and 19 rare variants was not statistically significantly associated with either DBP (*P* = 0.07) or SBP (*P* = 0.53). However, the joint effect of the 2 GWAS SNPs and 16 non-synonymous SNPs was significant for DBP (*P* = 0.03) but not for SBP (*P* = 0.41).

**Table 2:** Main effects of 31 common variants in *PCSK9* on blood pressure in GWAS^*^.

**#**	**Common SNPs**	**Location chromosome 1**	**A1^+^/A2**	**MAF**	**HWE *P*-value**	**β**	**SE**	***P*-value**
1	*rs1603717_A*	55733832	A/G	0.3	0.3			
	DBP					0.6	0.5	0.3
	SBP					−0.3	0.98	0.8
2	*rs12048828_C*	55735734	C/T	0.08	0.6			
	DBP					1.8	0.93	**0.05**
	SBP					0.5	1.7	0.8
3	*rs6698904_T*	55737077	T/C	0.03	0.6			
	DBP					−0.8	1.5	0.6
	SBP					−1.4	2.8	0.6
4	*rs6588553_C*	55737455	C/T	0.2	0.1			
	DBP					−0.6	0.6	0.3
	SBP					−1.5	1.1	0.2
5	*rs10443235_C*	55738353	C/T	0.4	0.6			
	DBP					0.2	0.5	0.7
	SBP					0.5	0.95	0.6
6	*rs12086676_C*	55738663	C/T	0.4	0.5			
	DBP					−0.6	0.5	0.3
	SBP					0.06	0.9	0.95
7	*rs12076372_A*	55738727	A/C	0.1	1			
	DBP					−0.5	0.7	0.5
	SBP					−1.7	1.3	0.2
8	*rs6657064_G*	55738918	G/C	0.3	0.7			
	DBP					−0.4	0.5	0.4
	SBP					−0.9	0.97	0.4
9	*rs2088487_T*	55739925	T/C	0.3	0.2			
	DBP					−0.5	0.5	0.3
	SBP					−0.5	0.95	0.6
10	*rs17101343_C*	55741315	T/C	0.06	0.2			
	DBP					0.02	1.05	0.98
	SBP					−2.0	1.9	0.3
11	*rs6588555_A*	55741377	A/G	0.1	0.06			
	DBP					0.1	0.8	0.9
	SBP					0.02	1.4	0.98
12	*rs12035319_T*	55742746	T/C	0.02	1			
	DBP					3.4	1.9	0.07
	SBP					1.9	3.4	0.6
13	*rs2047418_G*	55743099	G/C	0.2	0.3			
	DBP					−0.6	0.6	0.3
	SBP					−0.6	1.0	0.5
14	*rs1874776_C*	55743519	T/C	0.3	0.2			
	DBP					−0.8	0.5	0.1
	SBP					−0.7	0.96	0.5
15	*rs2047420_T*	55744429	T/G	0.4	0.5			
	DBP					−0.2	0.5	0.6
	SBP					−1.6	0.95	0.1
16	*rs4926675_A*	55744950	A/G	0.2	0.5			
	DBP					0.7	0.7	0.3
	SBP					−0.2	1.1	0.9
17	*rs11206551_A*	55744966	G/A	0.1	0.1			
	DBP					−0.2	0.8	0.8
	SBP					0.7	1.5	0.7
18	*rs12077573_G*	55746146	C/G	0.1	0.1			
	DBP					−1.1	0.8	0.1
	SBP					−2.0	1.4	0.1
19	*rs12071742_A*	55747825	A/G	0.4	0.08			
	DBP					0.5	0.5	0.3
	SBP					1.6	0.95	0.08
20	*rs17405810_C*	55748043	C/G	0.08	0.04			
	DBP					−0.05	0.9	0.95
	SBP					0.4	1.7	0.8
21	*rs9787035_C*	55748166	C/A	0.01	0.3			
	DBP					−1.0	1.0	0.6
	SBP					2.2	3.6	0.5
22	*rs17111836_T*	55748970	T/C	0.08	0.7			
	DBP					0.9	0.9	0.3
	SBP					1.6	1.7	0.3
23	*rs4927218_A*	55749649	A/G	0.05	0.6			
	DBP					−1.0	1.2	0.4
	SBP					1.5	2.1	0.5
24	*rs9730100_C*	55750600	C/A	0.4	0.4			
	DBP					1.0	0.5	**0.05**
	SBP					1.3	0.9	0.2
25	*rs4927219_A*	55751821	A/G	0.3	0.3			
	DBP					0.9	0.6	0.1
	SBP					1.1	1.0	0.3
26	*rs17111859_T*	55752440	T/C	0.2	0.5			
	DBP					−0.1	0.6	0.9
	SBP					1.3	1.1	0.2
27	*rs17111863_C*	55752896	C/T	0.09	1			
	DBP					0.8	0.9	0.3
	SBP					2.3	1.6	0.1
28	*rs11809596_A*	55753233	G/A	0.5	0.2			
	DBP					0.2	0.5	0.7
	SBP					0.9	0.9	0.3
29	*rs17111872_C*	55755376	C/G	0.1	0.6			
	DBP					0.8	0.7	0.3
	SBP					2.0	1.3	0.1
30	*rs17111875_C*	55757023	C/A	0.03	1			
	DBP					1.4	1.4	0.3
	SBP					3.2	2.5	0.2
31	*rs10888912_A*	55757807	A/G	0.3	0.5			
	DBP					0.4	0.6	0.4
	SBP					−0.3	1.0	0.8

* *Model adjusted for age, sex, center, smoking status, diabetes, and family random effect*.

^*^A1, minor allele; β, regression coefficient; DBP, diastolic blood pressure; GWAS, genome-wide association study; HWE, Hardy-Weinberg Equilibrium; MAF, minor allele frequency; SBP, systolic blood pressure; SE, standard error.

*Bold values signifies significant results at P ≤ 0.05*.

**Table 3:** ***PCSK9* rare variants in the exome chip data**.

**No**	**SNP**	**rs#**	**Location on chromosome 1**	**Nucleotide position**	**Protein position**	**Annotation**	**MAF**	**Number of carriers**	**HWE *P*-value**	**SBP median**	**DBP median**
1	exm62588	rs11591147	55505647	c.137 G>T	p.R46L	Non-synonymous	0.002	9	1	137	89
2	exm62591	rs145886902	55505679	c.169 G>A	p.E57K	Non-synonymous	0.002	8	1	133	66
3	exm62606	rs67608943	55512222	c.426 C>G	p.Y142X	Stop-gain	0.004	15	1	116	75
4	exm62607	Not available	55512267	c.471 C>A	p.N157K	Non-synonymous	0.0002	1	1	165	97
5	exm2263347	rs149489325	55518371	c.706 G>A	p.G236S	Non-synonymous	0.002	3	1	135	81
6	exm62636	rs72646508	55518422	c.757 C>T	p.L253F	Non-synonymous	0.003	14	1	128	75
7	exm62652	Not available	55523076	c.1069C>T	p.R357C	Non-synonymous	0.0002	1	1	122	75
8	exm62659	rs146471967	55523178	c.1171 C>A	p.H391N	Non-synonymous	0.002	10	1	138	82
9	exm62666	rs143275858	55523779	c.1251 C>A	p.H417Q	Non-synonymous	0.002	7	1	132	78
10	exm62667	rs28362261	55523802	c.1274 A>G	p.N425S	Non-synonymous	0.01	72	0.139	135	79
11	exm62678	rs141502002	55524222	c.1405 C>T	p.R469W	Non-synonymous	0.01	40	0.211	129	76
12	exm1834776	rs141995194	55524262	c.1445 A>G	p.E482G	Non-synonymous	0.002	6	1	130	80
13	exm62699	rs28362270	55525313	c.1658 A>G	p.H553R	Non-synonymous	0.01	55	0.3558	134	76
14	exm62700	rs140364657	55525315	c.1851 C>G	p.Q554E	Non-synonymous	0.001	6	1	135	78
15	exm62708	rs28362277	55527222	c.1856 A>C	p.Q619P	Non-synonymous	0.01	60	1	146	84
16	exm62720	rs28362286	55529215	c.2037 C>A	p.C679X	Stop-gain	0.009	34	1	129	76
17	exm62616	rs148612296	55517952	c.525 C>T	p.D175D	Synonymous	0.009	34	1	136	77
18	exm2252207	rs28385710	55518418	c.753C>T	p.R251R	Synonymous	0.0002	1	1	207	105
19	exm2252208	rs140364657	55527217	c. 1851 C>T	p.A617A	Synonymous	0.006	20	1	135	79

*DBP, diastolic blood pressure; HWE, Hardy-Weinberg Equilibrium; MAF, minor allele frequency; SBP, systolic blood pressure*.

In REGARDS data, 18 of 19 *PCSK9* SNPs from exome chip data (SNP “exm62667” did not pass QC in the REGARDS dataset) were available for our analysis. *PCSK9* rare variants had a cumulative significant association with SBP (*P* = 0.04) but not with DBP (*P* = 0.36). The results did not change when restricted to 15 non-synonymous SNPs (*P* = 0.04 for SBP and *P* = 0.40 for DBP).

## Discussion

Using existing exome chip and GWAS data from HyperGEN, we tested the association of *PCSK9* polymorphisms with blood pressure. We found a marginal effect of two GWAS SNPs (rs12048828 and rs9730100) and a significant cumulative effect of all *PCSK9* rare variants, driven by non-synonymous SNPs, on DBP. Though our results for the two GWAS SNPs and for DBP did not replicate, we found a cumulative association with SBP in REGARDS, suggesting that rare variants in *PCSK9* may be important for blood pressure regulation. Overall, the median blood pressure was higher by rare-variant status in our study suggesting that these variants may downregulate function (i.e., less degradation of ENaC); however we cannot draw conclusions based on our research and future studies are required to understand the functional impact of these variants.

*PCSK9* is located on chromosome 1p32.3. It has 12 exons and encodes a 692 amino acid glycoprotein belonging to the family of protein convertases (Benjannet et al., 2004). More than 53 non-synonymous variants and 17 synonymous variants associated with cholesterol metabolism have been identified (Abifadel et al., 2009). Additionally, the rs11206510 (Schunkert et al., 2011), rs2479409 (Willer et al., 2013), and rs2495478 (Turnbull et al., 2012) variants have been highlighted by previous GWAS of lipid traits. Despite the plethora of studies on *PCSK9* mutations in cholesterol metabolism (Abifadel et al., 2003), its role in the etiology of hypertension remains poorly understood. A recent animal study (Sharotri et al., 2012) showed that *PCSK9* could alter blood pressure by modulating sodium absorption through ENaC. In that study *PCSK9* degraded ENaC during trafficking to the cell membrane, an effect linked to decreased blood pressure. *PCSK9* degradation of LDL-R at the cell membrane surface increases cholesterol. However, the mechanism of action of *PCSK9* on degration of ENaC and LDL-R (during trafficking to the cell membrane vs. at the cell membrane, respectively) is different, and teasing out how these polymorphisms affect the function of *PCSK9* in relation to blood cholesterol and blood pressure was beyond the scope of this study. Recent evidence from a mouse model suggests that knocking out the *PCSK9* gene does not change BP or sodium balance in mice (Berger et al., 2015). Our findings are clearly discrepant in comparison to that report, which may be due to insufficient homology between humans and mice, statistical power, or chance.

In addition to animal studies, a recent report examined the association of *PCSK9* and eight other lipid-related gene polymorphisms with blood pressure in a Chinese population stratified by gender (Yin et al., 2012). They tested only one variant of *PCSK9*, rs505151 (E670G), and reported a correlation with DBP in both male and female hypertensive groups. However, rs505151 did not have significant effect on DBP in the HyperGEN sample (*P* = 0.37). To our knowledge, our study is the first to investigate the association of multiple common and rare *PCSK9* variants with blood pressure. Interestingly in our study the association of *PCSK9* was observed only with DBP in the HyperGEN cohort and only with systolic blood pressure in the REGARDS cohort. This discrepancy could be due to differences between the REGARDS and HyperGEN populations. REGARDS participants are on average older than HyperGEN participants 64 ± 9 vs. 46 ± 13. Mean SBP (131 ± 17 in REGARDS vs. 129 ± 22 in HyperGEN) and DBP (79 ± 10 in REGARDS and 74 ± 12 in HyperGEN) are slightly lower in HyperGEN and there is a higher prevalence of antihypertensive treatment in REGARDS (68% vs. 57%). Importantly, hypertension at younger ages is characterized by increased DBP. As the arteries age and become more atherosclerotic, SBP becomes the dominant hypertension trait (Chrysant, 2013). If *PCSK9* is truly associated with hypertension, then it is reasonable to speculate it would associate with DBP at younger ages and SBP at older ages.

The strength of our study is the inclusion of African Americans, a historically under-represented population in genomic studies. Moreover, the majority of our population is also obese and, thus, at high risk for cardiovascular disease. Also, our study is the first to examine the association of multiple variants in *PCSK9* with blood pressure. Our findings must also be interpreted in context of some limitations. First, our study does not capture all common and rare variants in *PCSK9*. Future sequencing studies of African-American populations are necessary to fully cover the gene. Second, as *PCSK9* variants are unequally distributed in different populations (Abifadel et al., 2009), our findings might not generalize to other ethnic groups.

On balance, our results support the emerging hypothesis that *PCSK9* rare variants may have an involvement in the regulation of blood pressure. Upon further validation, our results may provide additional evidence of the pleiotropic function of *PCSK9*. Future studies should capitalize on sequencing data to further support or refute these hypotheses.

### Conflict of interest statement

The authors declare that the research was conducted in the absence of any commercial or financial relationships that could be construed as a potential conflict of interest.
