# Supplementary material for: PCSK9 variation and association with blood pressure in African Americans: preliminary findings from the HyperGEN and REGARDS studies
Source: Front Genet. 2015 Apr 8;6:136. doi: 10.3389/fgene.2015.00136 (PMC4389541; doi:10.3389/fgene.2015.00136)
Supplement: Supplementary file 1 [file Table1.DOCX]

***Supplementary Material***

**PCSK9 variation is associated with blood pressure in African Americans: findings from the HyperGEN study**

**Ngan T. Tran^1^, Stella Aslibekyan^1^ , Hemant K. Tiwari^2^, Degui Zhi^2^, Yun Ju Sung^3^, Steven C. Hunt^4^, DC Rao^3^, Ulrich Broeckel^5^, Suzanne Judd^5^, Paul Muntner^1^, Shia T Kent^1^, Donna K. Arnett^1^, Marguerite R. Irvin^1*^.**

^1^Department of Epidemiology, University of Alabama at Birmingham, Birmingham, AL, USA

^5^Department of Biostatistics, University of Alabama at Birmingham, Birmingham, AL, USA

^3^Department of Biostatistics, Washington University in St. Louis, St. Louis, MO, USA

^4^Department of Internal Medicine, University of Utah, Salt Lake City, UT, USA

^5^Human and Molecular Genetics Center, Medical College of Wisconsin, Milwaukee, WI, USA

Supplementary Figure 1. Linkage disequilibrium (LD) structure of the 50 common PCSK9 SNPs in HyperGEN GWAS data. The SNPs are shown sequentially as they appear on the chromosome. Pairwise LD values [D'(coefficient of linkage disequilibrium)/logarithm of odds (LOD)], estimated for the genotyped SNPs, are represented by boxes: dark gray indicates strong LD, light gray uninformative, whereas white denotes strong evidence of recombination. D' values are shown within the boxes; empty cells are in complete LD.
