# Supplementary figures and images for: PCSK9 variation and association with blood pressure in African Americans: preliminary findings from the HyperGEN and REGARDS studies
Source: Front Genet. 2015 Apr 8;6:136. doi: 10.3389/fgene.2015.00136 (PMC4389541; doi:10.3389/fgene.2015.00136)

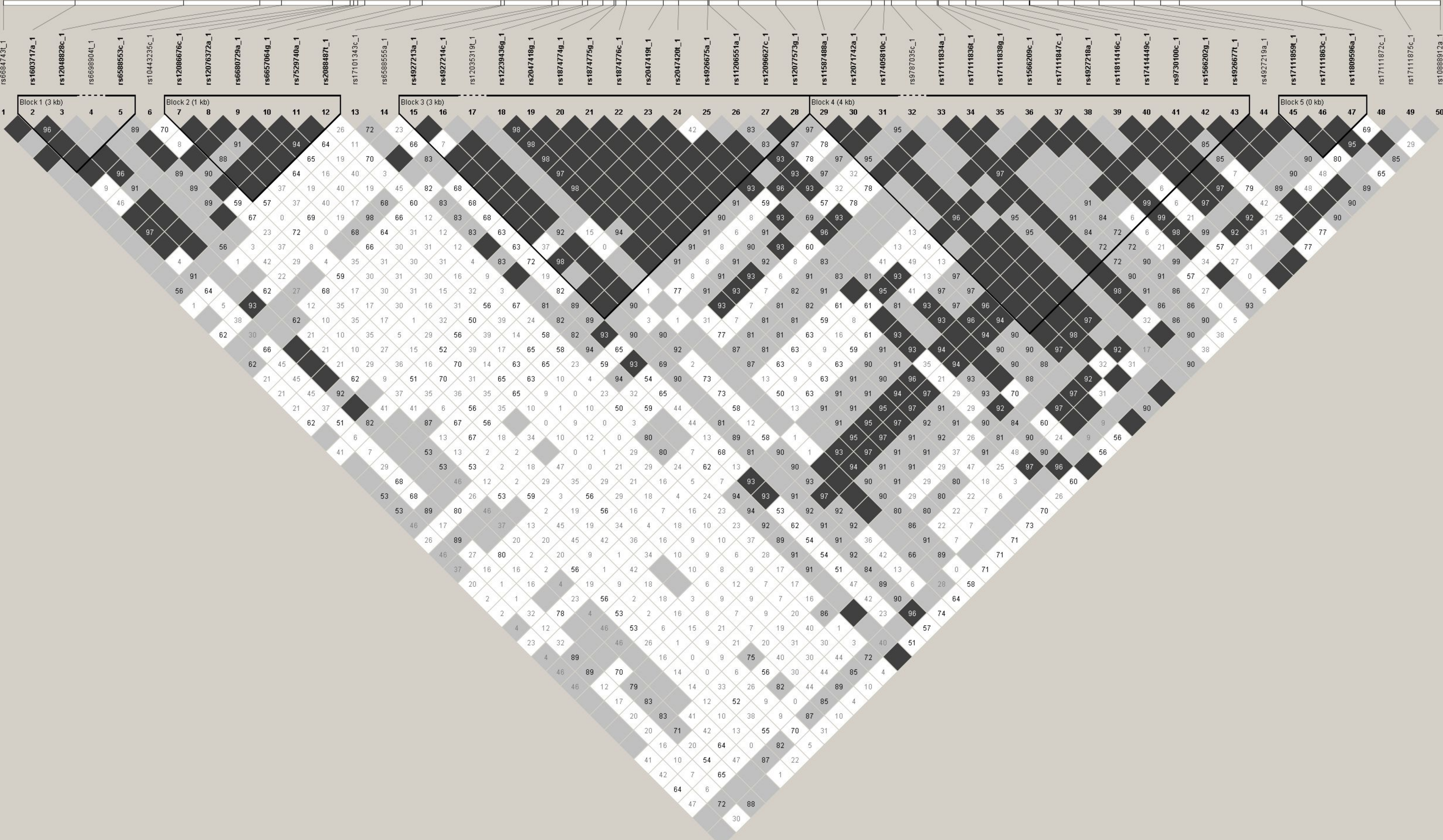

Supplement: Supplementary file 2 [file Presentation1.PDF]
